# The transcriptome data from the leaves of four *Papaver* species captured at the plant's three developmental life cycles

**DOI:** 10.1016/j.dib.2019.104955

**Published:** 2019-12-07

**Authors:** Sathiyamoorthy Subramaniyam, Seonhwa Bae, Myunghee Jung, Younhee Shin, Jae-Hyeon Oh

**Affiliations:** a Research and Development Center, Insilicogen Inc., Yongin-si 16954, Gyeonggi-do, Republic of Korea; b Genomics Division, National Institute of Agricultural Science (NAS), Rural Development Administration (RDA), 370, Nongsaengmyeong-ro, Wansan-gu, Jeonju-si, Jeollabuk-do 54874, Republic of Korea; c Department of Forest Sciences, College of Agriculture and Life Sciences, Seoul National University, Seoul 08826, Republic of Korea; d Department of Biological Sciences, Sungkyunkwan University, Suwon 16419, Republic of Korea; e Upland Crop Breeding Research Division, Department of Southern Area Crop Science, National Institute of Crop Sciences (NICS), Rural Development Administration (RDA), 20 Jeompiljae-ro, Miryang, Gyeongnam 50424, Republic of Korea

**Keywords:** Papaver, Transcriptome, Developmental stages, Alkaloids, Poppies

## Abstract

The plants in the Papaver genus are widely known as Poppies, which is used for ornamental and medicinal purposes, to utilize its plants derived alkaloids and attractive flowers. From this genus, we have sequenced the transcriptomes of four species's (*Papaver rhoeas* (two cultivar)*, Papaver nudicaule* (five cultivar)*, Papaver fauriei,* and *Papaver somniferum*) leaves at three developmental stages (i.e., leaf rosette (30 days), elongation and branching (60 days), and blossom and seed formations (90 days)), to elucidate the secondary metabolite biosynthesis gene expression profiles at respective plant stages.

**Table:** Specifications Table

Subject	Biology
Specific subject area	Transcriptomics
Type of data	Table, Figure
How data were acquired	Illumina Hiseq™ 4000
Data format	Raw sequences (FASTQ)
Parameters for data collection	Three developmental stages, i.e., leaf rosette (30 days), elongation and branching (60 days), and blossom and seed formations (90 days)
Description of data collection	Papaver plants were grown individually in multiple pots and maintained at 30 °C for 3 months. At three time points (30, 60, and 90 days (and 120 days for *P. fauriei* only)), individuals were selected for leaf samplings. The leaves collected for the transcriptome analysis have been frozen immediately in liquid nitrogen and stored in a deep freezer at −70 °C. For each species, the experiments were repeated in triplicates (under the same conditions).
Data source location	National Institute of Agricultural Science, Republic of Korea
Data accessibility	Raw data of the RNA-Seq are available on Sequence Read Archive (SRA) and it has been deposited at NCBI under the bioproject accession PRJNA476004 (https://www.ncbi.nlm.nih.gov/bioproject/PRJNA476004).

**Table:** 

**Value of the Data** • This transcriptome data can be useful to elucidate the transcriptome-wide association SNP markers and to assess the differences in the quantity of secondary metabolites, among and within Papaver species and subspecies. • The phenotypic data (Flower colour, petal arrangements, number of petals) can be useful to identify the associated SNP markers for more detailed characterizations. • The iso-seq data from two samples may help to improve the existing gene annotation of the representative *Papaver somniferum* genome.

## 1 Data

**Fig. 1:**
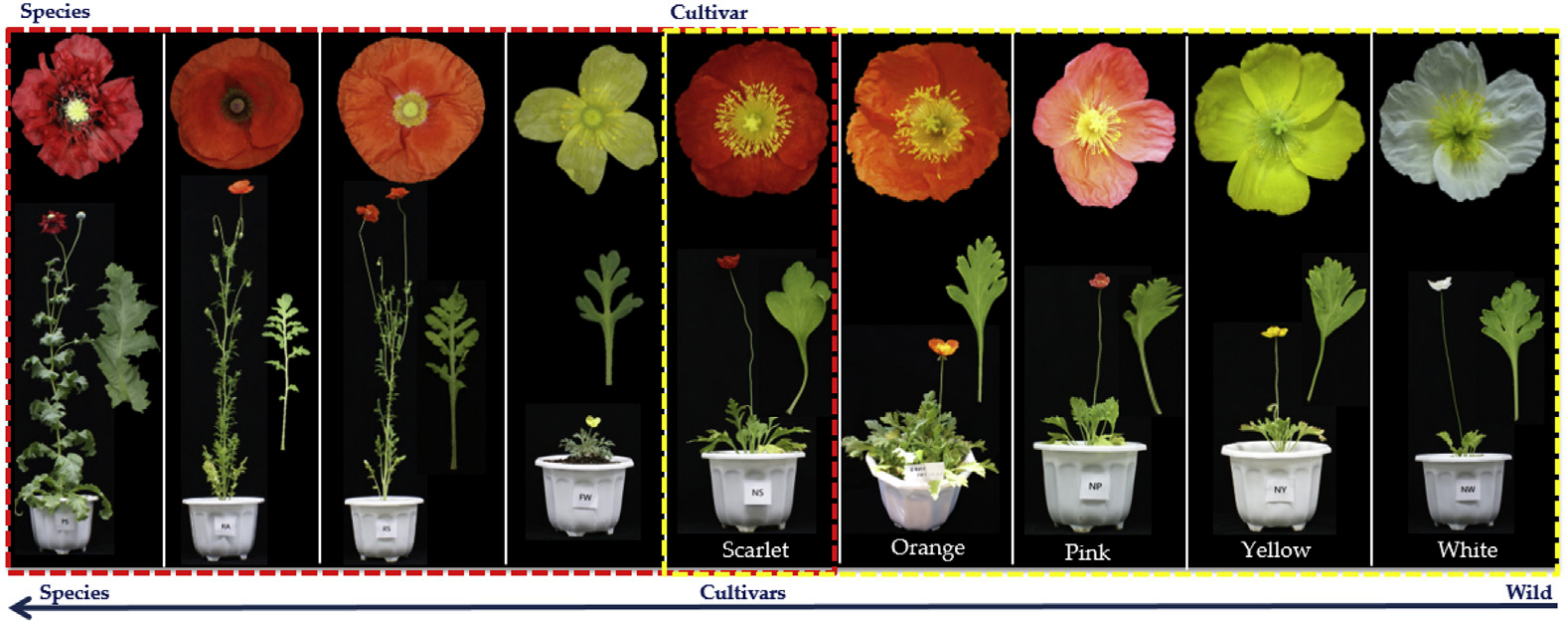
The morphological illustrations of Papaver species. The species from the right is *Papavar somniferum, P. rhoeas (Asia red A and B), P. fauriei and P. nudicaule. Papaver nudicaule* cultivars (yellow dotted lines) and different *Papaver* species (Red dotted lines).

The dataset present in this article is a transcriptome from the leaves of four Papaver species and its subspecies classified upon their flower colour, as shown in Fig. 1. The tables in this article are as follows: Table 1 explains the sampling time points of Papaver plant from its three different growth stages, and Table 2 explains the quality of the transcriptome data and the sequences mapped to the draft genome and the reference transcriptome. Totally, 590 Gb of transcriptome sequences are generated from 84 sequence libraries (i.e., 28 sampling points with three biological replicates) using Illumina Hi-Seq 4000 equipment and 481 Mb of long reads from 2 libraries using PacBio, iso-seq method. Among those, the short reads, 568.4 GB (96.2%) of bases remained after the pre-processing, as explained in the previous articles [1,2]. Complete reference transcriptome has been employed for the de-novo transcriptome assemblies, as explained in the previous articles [1,2]. Further, the pre-processed reads are mapped to the transcript references, which were obtained from the de-novo assemblies [1,2] and *Papaver somniferum* draft genome [3]. The coverage of sequence transcriptome is 77X per sample, which was calculated with the reference of transcripts obtained from the draft genome of *Papaver somniferum*. Part of this transcriptomic data was assessed to catalogue the available secondary metabolite biosynthesis transcripts and the cytochrome multi-family transcripts to the KEGG and cytochrome P450 engineering database (CYPED) [1,2]. Moreover, the differential expression profiles of those transcripts were assessed into two data models, i.e., between the stages of the developmental life cycle and between the Papaver species systematically [1,2]. Moreover, as the genome sequence has been utilized to explain the evolutionary history of morphine pathway [4], and to elucidate their core functions that exist in Papaver plant which can adapt to the whole plant community, as it is self-incompatibility to various environments [5]; hence, this data set could be valuable to assess the genetics behind the Papaver plant functions.

**Table 1:** Summary of the Papaver leaves sampled for the transcriptome sequencing.

Plant (ID)	Flower Color	Methods	Plant age in Days			
			30	60	90	120
*Papaver rhoeas* (RA)	Asia Red A	Illumina	✓	✓	✓	°
*Papaver rhoeas* (RS)	Asia Red B	Illumina/PacBio	✓	✓	✓	°
*Papaver nudicaule* (NW)	White	Illumina/PacBio	✓	✓	✓	°
*Papaver nudicaule* (NO)	Orange	Illumina	✓	✓	✓	°
*Papaver nudicaule* (NY)	Yellow	Illumina	✓	✓	✓	°
*Papaver nudicaule* (NS)	Scarlet	Illumina/PacBio	✓	✓	✓	°
*Papaver nudicaule* (NP)	Pink	Illumina	✓	✓	✓	°
*Papaver fauriei* (FW)	Yellow	Illumina	✓	✓	✓	✓
*Papaver somniferum* (PS)	Scarlet	Illumina	✓	✓	✓	°

**Table 2:** The sequence summary of individual samples. The reference are 1: Oh, J. et al., 2: Kim, D. et al., and 3: this article.

Given Name	Raw Bases	Processed (%)	Reference Mapping		Accession link	Ref
			Genome	Transcriptome		
FW_120_1	6.60	96.62	39.64	76.68	https://trace.ncbi.nlm.nih.gov/Traces/sra/?run=SRR8437644	2
FW_120_2	5.90	96.59	41.73	75.73	https://trace.ncbi.nlm.nih.gov/Traces/sra/?run=SRR8437645	2
FW_120_3	6.30	96.52	39.68	78.05	https://trace.ncbi.nlm.nih.gov/Traces/sra/?run=SRR8437646	2
FW_30_1	8.20	97.42	44.46	75.02	https://trace.ncbi.nlm.nih.gov/Traces/sra/?run=SRR8437675	2
FW_30_2	8.20	97.41	44.46	75.02	https://trace.ncbi.nlm.nih.gov/Traces/sra/?run=SRR8437674	2
FW_30_3	8.20	97.41	44.46	75.02	https://trace.ncbi.nlm.nih.gov/Traces/sra/?run=SRR8437671	2
FW_60_1	5.90	96.62	40.32	75.96	https://trace.ncbi.nlm.nih.gov/Traces/sra/?run=SRR8437670	2
FW_60_2	6.00	96.65	40.34	75.25	https://trace.ncbi.nlm.nih.gov/Traces/sra/?run=SRR8437647	2
FW_60_3	6.00	96.76	39.56	75.72	https://trace.ncbi.nlm.nih.gov/Traces/sra/?run=SRR8437648	2
FW_90_1	7.20	96.96	44.95	75.90	https://trace.ncbi.nlm.nih.gov/Traces/sra/?run=SRR8437639	2
FW_90_2	7.60	96.94	43.95	75.80	https://trace.ncbi.nlm.nih.gov/Traces/sra/?run=SRR8437642	2
FW_90_3	5.70	97.08	45.14	75.89	https://trace.ncbi.nlm.nih.gov/Traces/sra/?run=SRR8437643	2
NO_30_1	6.10	97.94	48.13	84.43	https://trace.ncbi.nlm.nih.gov/Traces/sra/?run=SRR8437690	3
NO_30_2	6.90	98.02	47.81	84.85	https://trace.ncbi.nlm.nih.gov/Traces/sra/?run=SRR8437689	3
NO_30_3	6.20	98.04	46.82	85.61	https://trace.ncbi.nlm.nih.gov/Traces/sra/?run=SRR8437657	3
NO_60_1	6.00	97.22	46.53	84.13	https://trace.ncbi.nlm.nih.gov/Traces/sra/?run=SRR8437658	3
NO_60_2	12.90	95.45	51.28	70.75	https://trace.ncbi.nlm.nih.gov/Traces/sra/?run=SRR8437655	3
NO_60_3	6.90	97.08	46.96	83.96	https://trace.ncbi.nlm.nih.gov/Traces/sra/?run=SRR8437656	3
NO_90_1	5.40	96.54	44.71	85.31	https://trace.ncbi.nlm.nih.gov/Traces/sra/?run=SRR8437653	3
NO_90_2	6.60	96.67	47.12	84.46	https://trace.ncbi.nlm.nih.gov/Traces/sra/?run=SRR8437654	3
NO_90_3	7.10	96.39	45.44	84.99	https://trace.ncbi.nlm.nih.gov/Traces/sra/?run=SRR8437651	3
NP_30_1	6.20	97.94	47.74	84.31	https://trace.ncbi.nlm.nih.gov/Traces/sra/?run=SRR8437640	3
NP_30_2	5.60	97.90	45.60	85.49	https://trace.ncbi.nlm.nih.gov/Traces/sra/?run=SRR8437687	3
NP_30_3	5.40	97.97	47.63	84.25	https://trace.ncbi.nlm.nih.gov/Traces/sra/?run=SRR8437686	3
NP_60_1	6.50	97.02	46.78	83.48	https://trace.ncbi.nlm.nih.gov/Traces/sra/?run=SRR8437685	3
NP_60_2	12.30	96.52	43.40	85.06	https://trace.ncbi.nlm.nih.gov/Traces/sra/?run=SRR8437684	3
NP_60_3	5.90	97.38	47.18	83.92	https://trace.ncbi.nlm.nih.gov/Traces/sra/?run=SRR8437683	3
NP_90_1	5.20	96.41	44.77	85.52	https://trace.ncbi.nlm.nih.gov/Traces/sra/?run=SRR8437682	3
NP_90_2	6.60	96.79	46.34	84.30	https://trace.ncbi.nlm.nih.gov/Traces/sra/?run=SRR8437681	3
NP_90_3	5.40	96.63	46.97	84.51	https://trace.ncbi.nlm.nih.gov/Traces/sra/?run=SRR8437680	3
NS_30_1	5.50	97.58	47.31	84.46	https://trace.ncbi.nlm.nih.gov/Traces/sra/?run=SRR8437652	2
NS_30_2	6.00	97.40	47.35	84.02	https://trace.ncbi.nlm.nih.gov/Traces/sra/?run=SRR8437649	2
NS_30_3	6.20	97.30	47.79	83.08	https://trace.ncbi.nlm.nih.gov/Traces/sra/?run=SRR8437650	2
NS_60_1	6.40	97.36	46.81	84.53	https://trace.ncbi.nlm.nih.gov/Traces/sra/?run=SRR8437677	2
NS_60_2	13.50	96.99	42.88	84.21	https://trace.ncbi.nlm.nih.gov/Traces/sra/?run=SRR8437676	2
NS_60_3	5.50	97.24	49.80	83.47	https://trace.ncbi.nlm.nih.gov/Traces/sra/?run=SRR8437679	2
NS_90_1	7.30	94.43	45.68	82.78	https://trace.ncbi.nlm.nih.gov/Traces/sra/?run=SRR8437678	2
NS_90_2	5.80	96.69	45.34	84.13	https://trace.ncbi.nlm.nih.gov/Traces/sra/?run=SRR8437673	2
NS_90_3	7.70	96.68	46.28	84.57	https://trace.ncbi.nlm.nih.gov/Traces/sra/?run=SRR8437672	2
NW_30_1	6.80	97.83	44.60	85.97	https://trace.ncbi.nlm.nih.gov/Traces/sra/?run=SRR7345734	3
NW_30_2	6.50	97.98	46.39	85.93	https://trace.ncbi.nlm.nih.gov/Traces/sra/?run=SRR7345737	3
NW_30_3	7.00	98.01	44.04	86.48	https://trace.ncbi.nlm.nih.gov/Traces/sra/?run=SRR7345738	3
NW_60_1	6.40	96.97	47.56	83.77	https://trace.ncbi.nlm.nih.gov/Traces/sra/?run=SRR7345735	3
NW_60_2	6.50	97.15	47.93	83.92	https://trace.ncbi.nlm.nih.gov/Traces/sra/?run=SRR7345736	3
NW_60_3	5.20	96.54	45.33	85.14	https://trace.ncbi.nlm.nih.gov/Traces/sra/?run=SRR7345741	3
NW_90_1	6.20	96.75	44.88	85.25	https://trace.ncbi.nlm.nih.gov/Traces/sra/?run=SRR7345742	3
NW_90_2	6.30	95.28	46.58	82.60	https://trace.ncbi.nlm.nih.gov/Traces/sra/?run=SRR7345739	3
NW_90_3	6.60	96.61	44.10	84.84	https://trace.ncbi.nlm.nih.gov/Traces/sra/?run=SRR7345740	3
NY_30_1	8.10	97.99	46.20	84.94	https://trace.ncbi.nlm.nih.gov/Traces/sra/?run=SRR8437636	3
NY_30_2	6.30	97.96	47.90	84.44	https://trace.ncbi.nlm.nih.gov/Traces/sra/?run=SRR8437635	3
NY_30_3	6.80	97.90	48.36	84.64	https://trace.ncbi.nlm.nih.gov/Traces/sra/?run=SRR8437638	3
NY_60_1	5.50	97.08	48.57	82.74	https://trace.ncbi.nlm.nih.gov/Traces/sra/?run=SRR8437637	3
NY_60_2	11.10	96.64	42.09	85.65	https://trace.ncbi.nlm.nih.gov/Traces/sra/?run=SRR8437632	3
NY_60_3	5.70	97.35	46.54	82.95	https://trace.ncbi.nlm.nih.gov/Traces/sra/?run=SRR8437631	3
NY_90_1	6.90	96.66	45.14	84.56	https://trace.ncbi.nlm.nih.gov/Traces/sra/?run=SRR8437634	3
NY_90_2	6.40	96.32	44.87	85.06	https://trace.ncbi.nlm.nih.gov/Traces/sra/?run=SRR8437633	3
NY_90_3	6.60	96.57	44.02	84.81	https://trace.ncbi.nlm.nih.gov/Traces/sra/?run=SRR8437641	3
PS_30_1	8.20	97.60	78.41	71.72	https://trace.ncbi.nlm.nih.gov/Traces/sra/?run=SRR8437692	1
PS_30_2	5.60	97.64	79.65	72.04	https://trace.ncbi.nlm.nih.gov/Traces/sra/?run=SRR8437669	1
PS_30_3	6.40	97.57	77.44	72.39	https://trace.ncbi.nlm.nih.gov/Traces/sra/?run=SRR8437668	1
PS_60_1	6.30	97.26	75.35	72.70	https://trace.ncbi.nlm.nih.gov/Traces/sra/?run=SRR8437691	1
PS_60_2	5.80	97.07	72.97	71.88	https://trace.ncbi.nlm.nih.gov/Traces/sra/?run=SRR8437695	1
PS_60_3	6.50	97.26	74.19	72.85	https://trace.ncbi.nlm.nih.gov/Traces/sra/?run=SRR8437667	1
PS_90_1	5.20	96.45	74.50	73.24	https://trace.ncbi.nlm.nih.gov/Traces/sra/?run=SRR8437688	1
PS_90_2	5.80	97.22	75.25	73.81	https://trace.ncbi.nlm.nih.gov/Traces/sra/?run=SRR8437693	1
PS_90_3	7.10	96.97	75.39	73.79	https://trace.ncbi.nlm.nih.gov/Traces/sra/?run=SRR8437694	1
RA_30_1	6.30	96.82	53.05	69.14	https://trace.ncbi.nlm.nih.gov/Traces/sra/?run=SRR8437661	2
RA_30_2	6.30	97.58	47.46	72.65	https://trace.ncbi.nlm.nih.gov/Traces/sra/?run=SRR8437664	2
RA_30_3	5.90	97.20	51.24	70.39	https://trace.ncbi.nlm.nih.gov/Traces/sra/?run=SRR8437660	2
RA_60_1	6.60	97.16	53.37	70.31	https://trace.ncbi.nlm.nih.gov/Traces/sra/?run=SRR8437659	2
RA_60_2	13.20	97.11	36.96	87.81	https://trace.ncbi.nlm.nih.gov/Traces/sra/?run=SRR8437663	2
RA_60_3	6.70	97.01	53.65	69.64	https://trace.ncbi.nlm.nih.gov/Traces/sra/?run=SRR8437696	2
RA_90_1	12.40	96.32	50.34	70.99	https://trace.ncbi.nlm.nih.gov/Traces/sra/?run=SRR8437666	2
RA_90_2	8.50	97.01	53.83	70.65	https://trace.ncbi.nlm.nih.gov/Traces/sra/?run=SRR8437665	2
RA_90_3	6.80	97.23	53.27	71.65	https://trace.ncbi.nlm.nih.gov/Traces/sra/?run=SRR8437662	2
RS_30_1	6.10	97.50	52.59	69.56	https://trace.ncbi.nlm.nih.gov/Traces/sra/?run=SRR7345727	1
RS_30_2	11.30	97.04	48.05	71.11	https://trace.ncbi.nlm.nih.gov/Traces/sra/?run=SRR7345728	1
RS_30_3	6.70	97.53	53.10	70.31	https://trace.ncbi.nlm.nih.gov/Traces/sra/?run=SRR7345725	1
RS_60_1	6.10	97.02	52.55	71.22	https://trace.ncbi.nlm.nih.gov/Traces/sra/?run=SRR7345726	1
RS_60_2	12.60	96.80	48.86	72.67	https://trace.ncbi.nlm.nih.gov/Traces/sra/?run=SRR7345731	1
RS_60_3	7.10	96.85	53.70	70.60	https://trace.ncbi.nlm.nih.gov/Traces/sra/?run=SRR7345732	1
RS_90_1	6.30	96.94	53.05	71.68	https://trace.ncbi.nlm.nih.gov/Traces/sra/?run=SRR7345729	1
RS_90_2	7.00	96.96	52.47	71.75	https://trace.ncbi.nlm.nih.gov/Traces/sra/?run=SRR7345730	1
RS_90_3	7.70	96.64	52.27	70.35	https://trace.ncbi.nlm.nih.gov/Traces/sra/?run=SRR7345733	1
NS_Leaf^a^	0.24	100.00	–	–	https://trace.ncbi.nlm.nih.gov/Traces/sra/?run=SRR7345724	2
RS_Leaf^a^	0.23	100.00	–	–	https://trace.ncbi.nlm.nih.gov/Traces/sra/?run=SRR7345723	1

a Pacbio transcriptome sequence libraries.

## 2 Experimental design, materials, and methods

### 2.1 Plant samples

Five *Papaver nudicaule* varieties with different colours of flowers, i.e., white, yellow, pink, orange, and scarlet have grown individually in multiple pots and maintained at 30 °C for 3 months. For the mRNA sequencing, leaf samples were obtained from three developmental stages (i.e., 30, 60, and 90 days). Another four Papaver species (i.e., *P. rhoeas*, *P. nudicaule, P. somniferum, and P. fauriei*) have been sampled with a similar procedure that belongs to this project [1,2]. The samples collected for transcriptomic analysis was immediately frozen in the liquid nitrogen and stored in a deep freezer at −70 °C. For each species, the experiments were repeated in triplicates (under the same conditions). Phenotypic differences among these plants, i.e., flower colour, leaves, and the visual appearance of the plant with flowers, are shown in Fig. 1.

### 2.2 Transcriptome sequencing

The complete sequence library preparation and sequencing experiments for the Illumina protocols were conducted by Macrogen Inc. (Seoul, Korea) (http://www.macrogen.com), the authorized sequence service providers for every individual sample. Illumina Hi-Seq 4000 system has been used to sequence all the individual samples. The details on the RNA library construction was given in the published articles [1,2]. Total raw Illumina short reads from each sample underwent the pre-processing steps, in order to remove the adapter, and low-quality reads using Trimmomatic v0.36 [6]. The processed short reads were then mapped to the assembled transcriptome using Salmon v0.9.1 [7].

### 2.3 Dataset

The complete sequences generated in this article have been submitted to the GenBank sequence read archive (SRA) under the bio-project ID PRJNA476004, as given in Table 2.
